# Microstructural Characterisation and Wear Behaviour of Diamond Composite Materials

**DOI:** 10.3390/ma3021390

**Published:** 2010-02-24

**Authors:** James N. Boland, Xing S. Li

**Affiliations:** CSIRO Exploration and Mining, PO Box 883, Kenmore QLD 4069, Australia; E-Mail: xing.li@csiro.au (X.S.L.)

**Keywords:** diamond composite, superhard materials, wear rate, cutting brittle material, Raman, XRD, cathodoluminescence

## Abstract

Since the initial research leading to the production of diamond composite materials, there have been several important developments leading to significant improvements in the properties of these superhard composite materials. Apart from the fact that diamonds, whether originating from natural resources or synthesised commercially, are the hardest and most wear-resistant materials commonly available, there are other mechanical properties that limit their industrial application. These include the low fracture toughness and low impact strength of diamond. By incorporating a range of binder phases into the sintering production process of these composites, these critically important properties have been radically improved. These new composites can withstand much higher operating temperatures without markedly reducing their strength and wear resistance. Further innovative steps are now being made to improve the properties of diamond composites by reducing grain and particle sizes into the nano range. This review will cover recent developments in diamond composite materials with special emphasis on microstructural characterisation. The results of such studies should assist in the design of new, innovative diamond tools as well as leading to radical improvements in the productivity of cutting, drilling and sawing operations in the exploration, mining, civil construction and manufacturing industries.

## 1. Introduction

Diamond is one of the allotropic forms of carbon [1,2] and is renowned for its outstanding physical, chemical, electrical and mechanical properties. It is also prized for its brilliance, which has led to being exploited for both its scientific applications and its unique, socially accepted form in the jewellery market. On the industrial front it is remarkable to note that in the early usage of diamonds for mineral exploration applications, large carat-sized pieces of diamond were bonded into the crown of drill bits [3].

Diamond composite tools should be considered as a multi-component system requiring the successful performance of many subsystems. These subsystems include: (a) the diamond grains themselves, (b) the binder phase or phases used in the sintering operation to form the composite and (c) the method of bonding the composite element into the body of the tool. As cutting elements, these composites are subjected to severe abrasive/erosive wear regimes leading to the generation of high temperatures. So not only is the wear resistance of the composite critical to its operational performance but equally important is its thermal stability, thermal conductivity, impact resistance, thermal fatigue limit and fracture toughness. These properties determine the suitability of diamond composite materials for cutting, drilling and sawing operations in the mining and civil construction industries.

A wide-ranging study has been undertaken by the SMART*CUT team of researchers at CSIRO’s division of Earth Science and Resource Engineering and the Flagship Minerals Down Under. Over the past 15 years, the wear behaviour of hard and superhard materials has been researched with particular emphasis on the cutting of hard and abrasive rocks. Initially, the research concentrated on the abrasive wear behaviour of thermally stable diamond composite (TSDC) cutting elements with some minor studies of the performance of cubic boron nitride (cBN) and cemented tungsten carbide. However, with the increasing importance of tools manufactured with a polycrystalline diamond composite (PCD) coating on a WC substrate, the results of wear tests on these tools are also included in this review. These studies are restricted to tools manufactured using the high pressure, high temperature (HPHT) processes, although it is acknowledged that chemical vapour deposition (CVD) coatings of diamond on WC and other superhard carbide materials play a major part of the new-age diamond tooling industry.

This review covers the latest research results pertaining to materials characterisation techniques that can be profitably exploited in the study of the microstructural features in a wide range of commercially produced diamond composites. Further, the results of a simple, low cost abrasive wear testing method for superhard diamond composites is presented, leading to an empirical measurement of the wear rate of these composites. Based on these results, the wear rate expressed as the weight loss of the cutting element per unit volume of material cut is proposed as a quality factor for assessing the behaviour of these diamond composite cutting elements. Using the newly developed solid TSDC and diamond composite coated WC cutting elements, the micromechanics of the cutting of brittle materials such as stone and rocks has necessitated the development of a more general phenomenological model of the macro chipping processes.

## 2. Diamonds

A great deal of research has already been undertaken to understand the properties and defect structures of both natural and synthetic diamond grains with excellent coverage given in the following references [4,5,6,7]. What emerges from these fundamental studies is that the properties of both natural and synthetic diamonds can vary significantly and the choice of the most appropriate source material for tool manufacture depends on the application to which diamonds will be put. Nearly all the research referenced in this review paper will relate to synthetic diamonds with only an occasional referral to naturally occurring diamonds.

There are three main diamond manufacturing process: (1) high pressure, high temperature (HPHT) synthesis, usually operating in the diamond stability field [2,8], (2) chemical vapour deposition (CVD) [9,10] and (3) detonation synthesis [11]. Three important characteristics of diamond grit/grain used in composite manufacture are: (1) grain size, (2) grain shape and (3) strength [13]. Other physical properties that have important implications for the performance of diamond composites are: thermal stability, thermal fatigue strength, fracture toughness and impact strength. As these properties have been reviewed elsewhere [4,5,6] they will only be referred to indirectly whenever they relate to the wear behaviour of diamond composites.

## 3. Development of Diamond Composites

Diamond composites have a wide range of compositions and applications. They have been variously referred to as PCD (polycrystalline diamond), PDC (polycrystalline diamond compact/cutter) and TSP (thermally stable polycrystalline diamond composite, sometimes represented as TSDC). Although not commonly referenced as diamond composite material, diamond impregnated metal matrix composites (MMC) are an integral part of the industrial diamond industry. The common parameters to be considered for this wide range of diamond composites are: the quality of the diamond itself, the strength of the bonding between the diamond grain and the matrix, the diamond concentration in the composite and the wear rates of the diamond and matrix as a function of the abrasivity of the material being drilled, machined and sawn.

The quality of individual diamond grains used in the production of these composites will depend on the source material. Synthetic diamonds, the most commonly used variety, are produced under HPHT conditions and their quality will depend on the solvent-catalyst system used in production of the diamonds. The usual catalysts are Ni, Fe, Co, Mn and Ti [8]. These elements can be incorporated into the diamond structure to produce a wide range of point defects that have been extensively studied [5]. While such point defects are not expected to significantly influence the mechanical and wear properties of diamond, if their concentration is too high, they can aggregate into fine-scale nanoprecipitates. The coefficient of thermal expansion of these nanoprecipitates will be different from that of diamond itself so thermally induced residual stresses will be generated in these diamond grains by thermal cycling events during cutting. These stresses may well be sufficient to initiate microcracks which will have a detrimental effect on the strength, wear resistance and fatigue properties of the diamond grains. One outstanding exception to the apparent neutral role of point defects has recently been reported with boron-doped diamond crystals [15]. Thermal stability, compressive strength and impact toughness all showed significant increases in the doped crystals compared with the normal diamonds. The solvent/catalyst was based on Fe-Ni alloy and the HPHT conditions were given as 1300–1400 °C and 5.4–5.7 GPa, which is in the diamond stability field of the carbon phase diagram.

In addition to their being several manufacturing processes, the form and shape of the product grains from these manufacturing processes can vary widely. In the case of PCD and PDC, the actual diamond composite is normally moulded onto a substrate during sintering and, in most applications, the substrate is cemented tungsten carbide, referred to as WC in this review. The major advantages of this product format are (a) the binder phase in both components is cobalt and (b) in this hybrid form, the PCD element can be easily brazed onto any shaped tool. The obvious disadvantage is that such tooling components are limited to low-temperature conditions as the cobalt is a catalyst for the back transformation of diamond to graphite [16,17].

TSDC components are produced by either using silicon carbide as binder or leaching the unwanted cobalt component from the surface layers of the standard PCD. The earliest known product in the category was de Beer’s (now Element 6) Syndax 3 [18]. It was applied by Asahi Diamond in its hybrid core bits [12]. Despite the higher thermal stability of this material (stable to 1200 °C) early production samples were reported to be unsuitable because of their lower strength and wear resistance than PCD. Nevertheless the fracture toughness was twice that of diamond single crystals [14]. Because residual silicon was observed in these samples, it can be concluded that the reactive bonding-sintering process did not proceed to completion.

**Figure 1:**
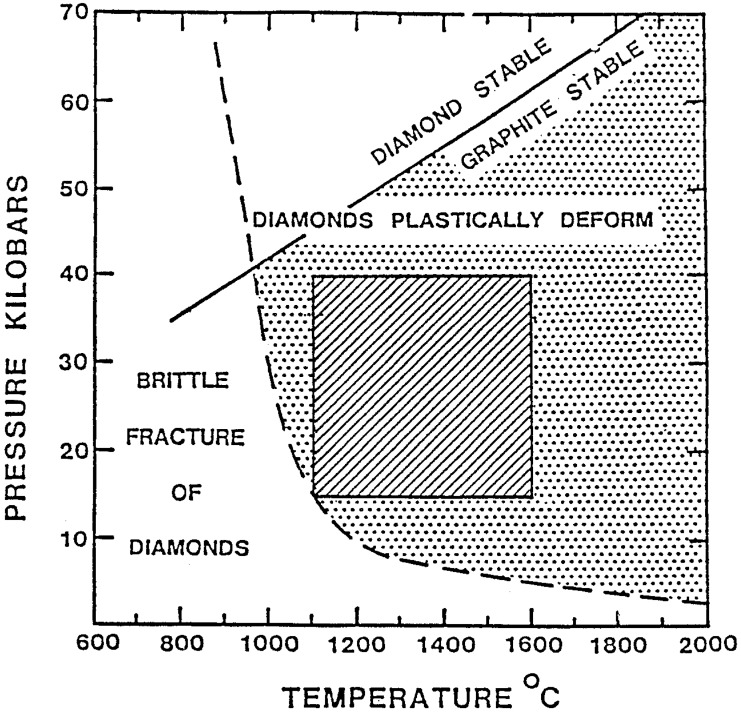
P-T phase diagram for carbon relevant to diamond composite manufacture. Note: 10kb = 1 GPa (Copy from US Patent: 4,948,388, 1989).

A new composite material was patented by A.E. Ringwood based on research in the mid 1980s [19]. This was known as ADC (advanced diamond composite). It was originally produced from natural diamond grit at pressures of about 2–2.5 GPa and at temperature ~1500 °C which is outside the diamond stability field and in the graphite phase field, Figure 1 [20].

Under these HPHT conditions the silicon metal melted and infiltrated the interstices of the granular structure of diamond grit. The silicon reacted with the diamond to form the refractory ceramic SiC which enabled strong bonding to occur between the diamond grains. To achieve optimum performance of the composite, the reactive sintering process should proceed to completion leaving no residual silicon metal in the microstructure. Another essential stage in the process is to rapidly quench the sintered product in order to avoid any back transformation of the diamond phase to graphite. The outstanding property exhibited by this type of composite is that it produces one of the earliest examples of thermally stable diamond composite–TSDC. Further, the microstructure indicated a high proportion of diamond-to-diamond grain boundary bonding. Such ceramic-type grain boundary bonding (equivalent to metallurgical bonds in metallic systems) produces a high contiguity value which is known to result in good mechanical properties in carbide systems [21,22]. The importance of microstructural features in diamond composites is given greater coverage in the next section.

The use of TSDC appears to be limited in the industry despite its potential to enable a much wider range of applications in the mining and manufacturing industries. The major issue to be addressed in the application of TSDC is the difficulty of bonding these cutting elements to a suitable substrate. Active brazing compounds, especially those containing Ti, are recommended to ensure strong bonding. The brazing has to be performed either at high vacuum or in an inert environment [23,24]. A simpler, less expensive bonding process has recently been patented, overcoming this difficulty in the usage of TSDC cutting elements in rock cutting picks and saw blades [25].

The major diamond composite type used in mining and manufacturing operations is PCD/PDC. An excellent history of the development of these diamond composites is given by Scott in [26]. PCD cutters have been shown to perform well in both civil construction and mining/exploration industries [27,28,29].

The physical properties of these composites are markedly dependent on both grain size and grain shape. In ceramic-type materials, the relationship between strength and grain size is complex. With PCD, being ceramic-like, it is not surprising to observe that the rupture strength is inversely proportional to d^1/2^ over the size range 2–30 microns [30]; for grain sizes greater than 30 microns, the strength drops off rapidly. The relationship between fracture toughness and grain size is less clearly defined but the normal trend, within the same grain size range, is an inverse relationship.

A major innovative step was taken with the patenting of nanostructured diamond-SiC composites [31,32]. This composite, based on at least one of the phases being in a nano grain size format, has produced excellent fracture toughness which both contradicts the usual inverse relationship between hardness and toughness and results in a product with toughness close to that of WC [13]. In this nanocomposite, the SiC phase was reported as being in the nano size range. Nanodiamonds are now being produced via a number of processes such as detonation, CVD and HPHT and they offer an opportunity for further research into the outstanding properties of nanocomposites.

## 4. Microstructure of Diamond Composites

From the outset, diamond composite materials present some challenges in undertaking detailed studies of their microstructures. Because they are superhard, sample preparation needs careful attention. Recently, a series of papers have been published detailing a successful approach to this issue [35]. Clearly, the better the surface preparation the more clearly defined the microstructural features become. However, not all surfaces need to be polished and this situation pertains particularly to the examination of worn surfaces in which it is essential to study the spatial distribution and near-neighbour relationships of all the phases exposed on that surface.

### 4.1. Observation techniques

A detailed study of the microstructures of diamond composites is essential to a proper understanding of their physical properties. It is important to note that this study has been undertaken on commercially available diamond composite products using optical and scanning electron microscopy (SEM equipped with energy dispersive system - EDS), electron probe microanalytical analysis (EPMA equipped for cathodoluminescence (CL), wavelength (WDS) and energy dispersive systems) with additional observations being made using micro-focus X-ray shadow imaging, X-ray diffraction (XRD) and Raman spectroscopy (RS). The surface preparation of samples for SEM imaging was varied. In some instances polished surfaces were studies but equally important for understanding the micromechanics of fracture and wear, are studies of as-fractured or as-worn surfaces.

### 4.2. Results

Thermally stable diamond composites (TSDC) have a wide range of uses apart from cutting elements. However, only those composites recommended for cutting have been studies in any detail and this review will concentrate on composites used mainly in cutting brittle materials such as rocks. The dominant phase in TSDC is diamond with a matrix of silicon carbide. Unless otherwise indicated, the specific TSDC samples obtained from the various manufacturers referred to in this review have a nominal 80 percent diamond and 20 percent binder matrix (by volume). Being thermally stable, the matrix is supposed to be SiC produced during the reactive bonding process at HPHT conditions in the diamond stability field—see Figure 1. While variations to this compositional mix will become obvious in the results presented in this review, the responsibility for these variations rests with the suppliers and such variations can be interpreted as issues of quality assurance which is outside the control of researchers.

**Figure 2:**
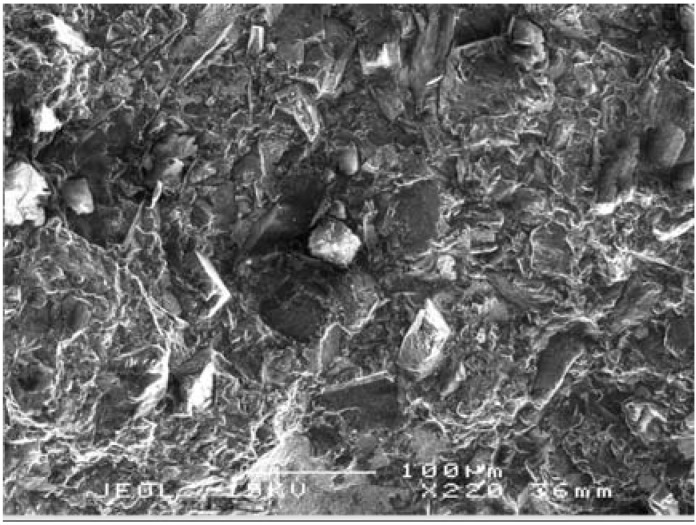
SEM images of TSDC cutting elements; (left) SEI of fracture surface with no evident pluck-out of diamond grains; (right) BEI of a polished surface with surface relief masking detailed phase information in the image.

As shown in Figure 2 (left), the fracture surface as viewed in the secondary electron image mode (SEI) indicates that the diamond grains have bonded to the matrix with no evident pluck-out of the diamond grains. With a moderate polish, the surface details are better viewed in the backscattered electron image mode (BEI) with the contrast arising from differences in the mean atomic number of the phases. The TSDC sample in Figure 2 (right) has a bi-modal grain size distribution but the polish is not sufficiently relief-free, with the surface roughness masking the complex phase distribution in the matrix.

**Figure 3:**
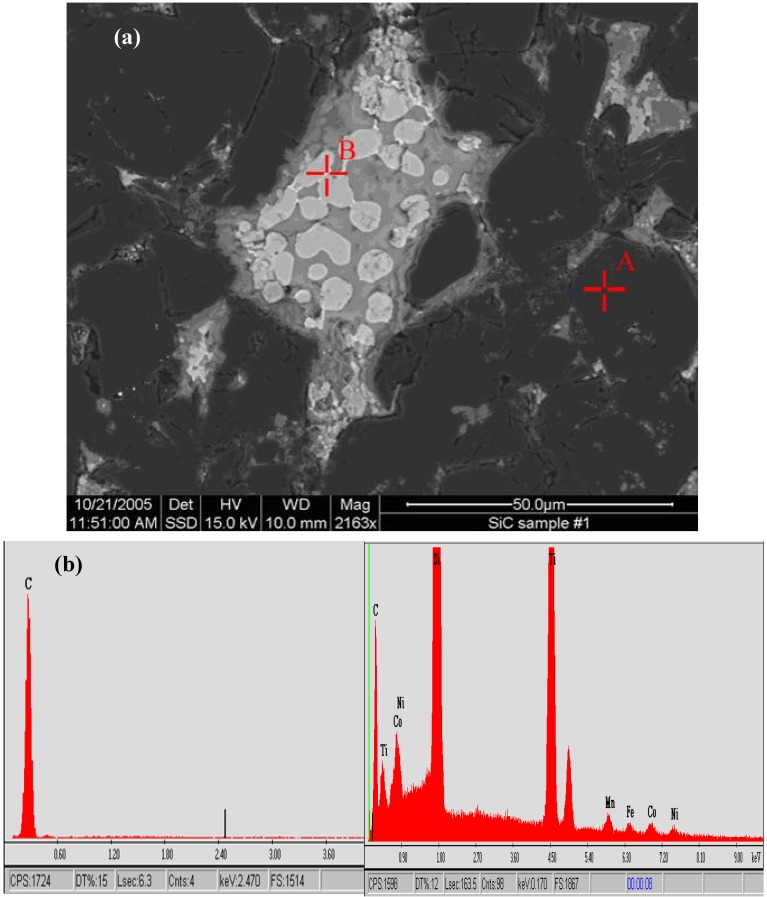
(a) SEM-BEI image of a TSDC sample showing the dominant diamond phase (A) and the complex, multi-phase matrix material. (b). EDS spectra from points A (left) and B (right). The single carbon peak at A clearly indicates the diamond phase; the multi-elemental spectrum analysed at point B clearly highlights the complexity of the matrix material in this commercially produced TSDC sample.

**Figure 4:**
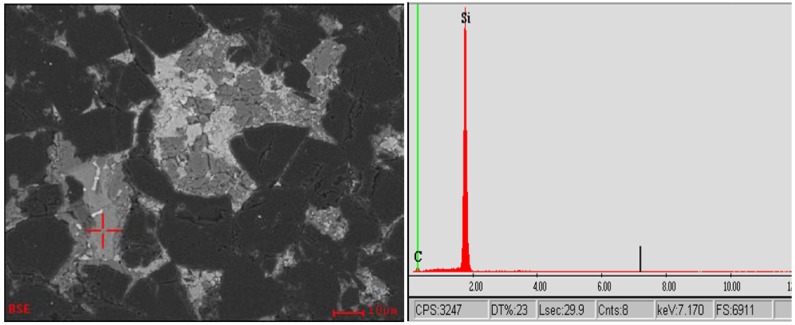
SEM-BEI image (left) from a TSDC sample with the corresponding EDS spot analysis of the light-grey phase indicating residual, unalloyed Si metal in the matrix.

**Figure 5:**
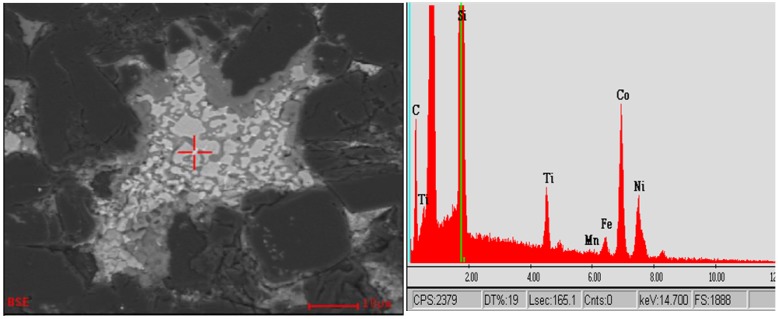
SEM-BEI image (left) from TSDC sample with the EDS spectrum (right) from the designed spot.

**Figure 6:**
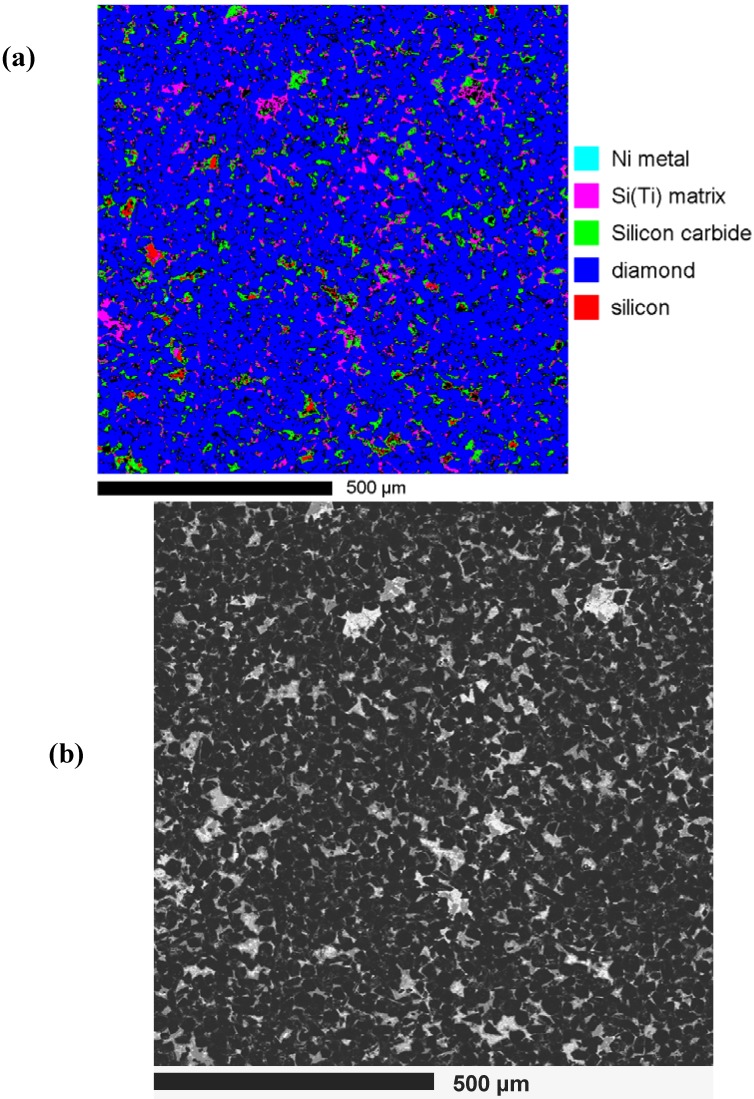

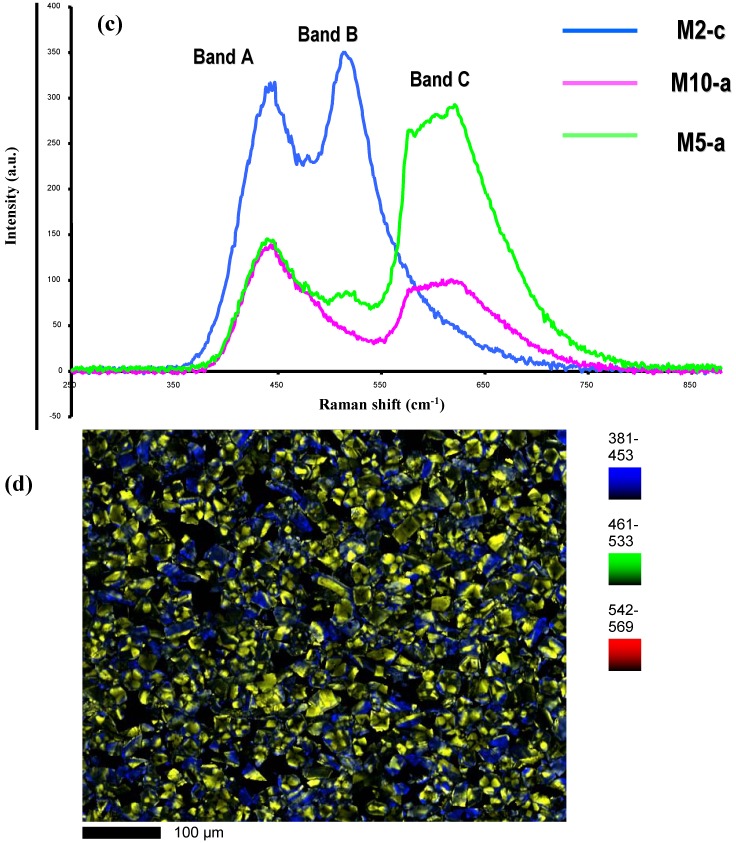
(a). SEM-BEI image of a TSDC sample. The dark grains are diamond and the various shades of lighter contrast in the matrix regions indicate the presence of several phases. (b). Hyperspectral image of the same field-of-view as in (a) in which the specific elements have been colour-rendered according to the attached colour table—see text for details. (c). Cathodoluminescence from TSDC samples listed in Table 1 showing room temperature CL spectra and the three prominent bands A, B and C. Note: M10-a and M3-f as shown in Table 1 are equivalent samples with respect to their wear resistance. (d). Mapping of the cathodoluminescent signals from a TSDC sample with the colour code showing the spectral range in nm—see text for details.

Using better polished surfaces, more specific microstructural and phase details can be observed. The following SEM-based microstructural images in Figure 3, Figure 4, Figure 5 and Figure 6 are from the same commercially produced TSDC sample. As the contrast in the BEI mode of imaging is related to variations in the mean atomic number of the phases, it is clear that there is a plethora of phases in the matrix interstitial areas. The determination of a particular phase may be simple as in the case of the single carbon peak in the EDS spectrum at point A in Figure 3 which could result from either graphite or diamond. Similarly, the single Si peak at the marked spot in Figure 4 indicates residual silicon metal in the TSDC. However, it has not been possible to identify the other minor phase constituents with the more complex elemental composition identified in the EDS spectra shown in Figure 3 (point B) and Figure 5. The presence of residual, unreacted silicon metal and/or graphite in TSDC samples has disastrous consequences for the wear behaviour of such materials as discussed in Section 5.

While this chemical information is useful in deciphering the possible phases at specific locations, the distribution of these phases can be analysed using X-ray mapping. However, a much more powerful approach to this issue is provided by combining the signals from several detectors attached to the SEM or probe. This patching together of detector signals has been referred to as hyperspectral or holistic mapping [36,37]. Examples of hyperspectral mapping are shown in Figure 6. The BEI image in Figure 6(a) shows similar image contrast effects to that presented in Figure 3, Figure 4 and Figure 5. By mapping signals from the various detectors via a colour code (or colour table), a modified SEM image is produced. It is now possible to identify distinct phases and their distribution in the sample as shown in Figure 6(b). For example, in this colour rendered image the CL active phases—diamond and SiC—and the characteristic X-ray photons for specific elements such as Ni, Si (metal) and a Si-Ti alloy have been colour coded, making it possible to identify the presence of residual silicon and a Si-Ti alloy as well as the reactively produced SiC phase. Additionally, small patches of Ni metal have been identified in this image.

From previous studies the zero-phonon lines in diamond have been identified [5]. Within the wavelength range 350–800 nm CL spectra have been recorded for the wide range of TSDC samples used in this study. Three dominant bands have been noted: band A with a peak at ~447 nm, band B with a peak at ~517 nm and a broad band C ranging from 576–626 nm—see Figure 6(c). Again, by mapping these peaks via the specified colour table, an image of the distribution of these CL active regions in the diamond can be identified—see Figure 6(d). This particular sample of TSDC is dominated by bands A and B and no evidence for band C. Band B has been previously identified with Type Ib synthetic diamond in which pure Co was the solvent-catalyst [5].

**Figure 7:**
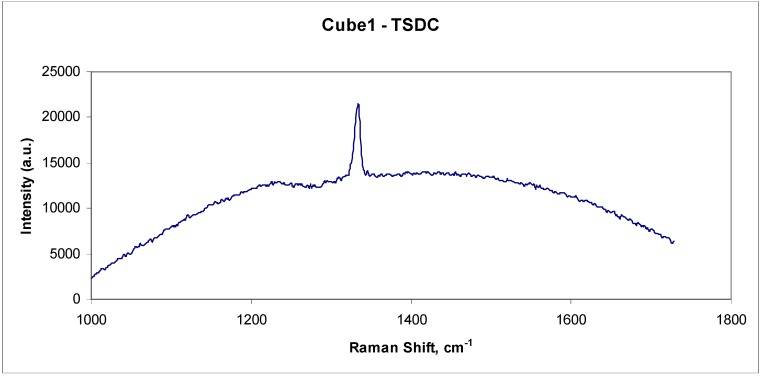
Raman spectrum from a good quality (low wear rate or high wear resistance) TSDC sample with no evidence of graphitic material.

Another valuable optical technique used to study diamond composite materials is Raman spectroscopy. This technique is capable of examining the finer details in the microstructure of the composite material by focusing a 2 μm laser beam onto any selected microstructural feature. It is capable of readily distinguishing between the allotropic forms of carbon. For example, diamond has a unique Raman peak in the spectrum at 1332 cm^-1^ and a typical spectrum from a diamond grain in a good quality (equivalent to high wear resistance) TSDC sample is shown in Figure 7. The spectrum from graphitic material is different, with broad peaks centred around 1350 and 1550 cm^-1^ and no peak at 1332 cm^-1^. These graphite peaks are referred to as the D and G modes (or bands) of disordered carbon [39].

**Figure 8:**
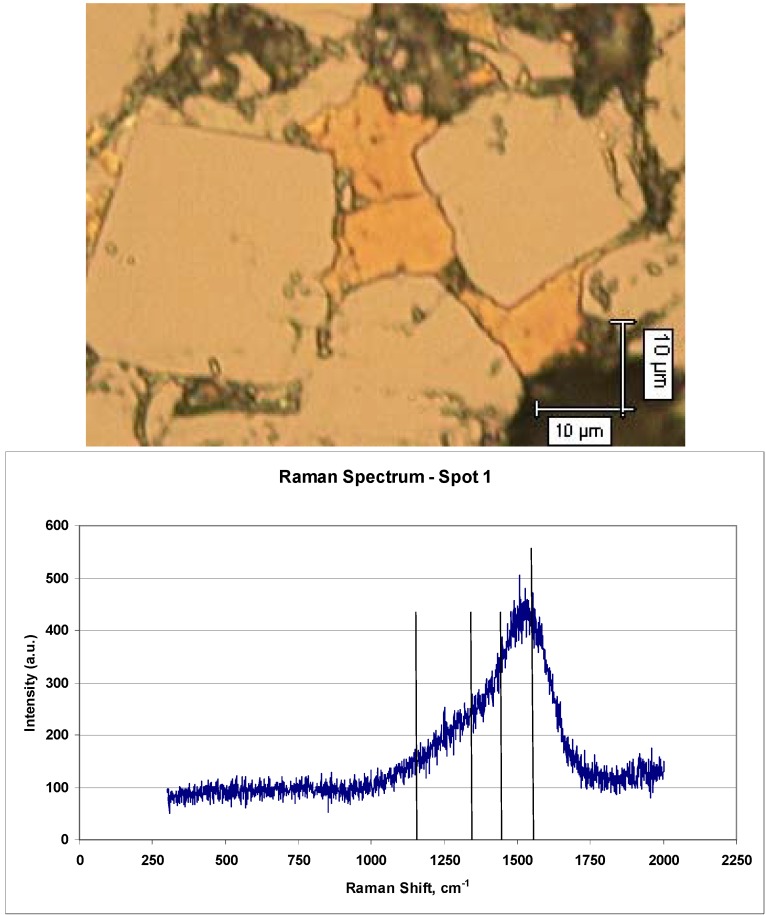
Optical micrograph (top) of poor quality (low wear rate or high wear resistance) TSDC material showing large (~25 μm), diamond grains with smaller (~10 μm) golden coloured grains with the distinctive graphitic Raman spectrum (bottom).

The optical micrograph in Figure 8 shows two different grain types, as indicated by their different reflectivities, within a poor quality (low wear resistance) TSDC sample. The larger (~25 μm), euhedral grains with the high reflectivity are the diamond phase but the smaller (~10 μm) grains exhibit a totally different Raman spectrum as shown in Figure 8. The broad peak centered around 1520 cm^-1^ is typical of graphitic material and is most likely associated with the D and G bands of disordered carbon. Further, there is no evidence of the diamond peak at 1332 cm^-1^.

Raman signals may also be used to produce hyperspectral images or chemical images of a sample in a manner not too dissimilar from the images created in EPMA—see Figure 6(c). Such microspectroscopy with a spatial resolution of the order of 2 μm would be a valuable aid in detecting diamond and graphitic phases in TSDC cutting elements.

Finally, standard X-ray diffraction or XRD can be used to determine the phases in TSDC samples but it has limitations due to the low detectability limits of the minor phases. A major advance in this technique has been achieved with the use of micro-XRD systems, especially on beamlines at synchrotron sources. With the high brilliance and small spot sizes available in this technique several square millimetres of sample can be quickly scanned to determine the phases in materials. A more macroscopic approach to XRD is given in the next section.

## 5. Wear Behaviour of Diamond Composite Tooling

**Figure 9:**
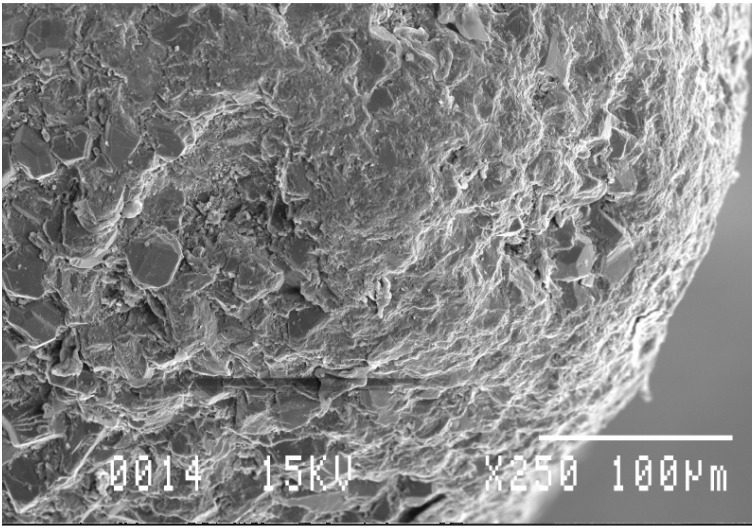
SEM-SEI image of the wear surface of a TSDC cutting element subjected to the standard pin-on-disk abrasive wear test.

Diamond composite along with cubic boron nitride form the group of superhard, abrasive resistant materials that have great potential to revolutionise the cutting, drilling and sawing operations in the mining and civil construction industries. Because of their extreme wear resistance, standard wear testing procedures [42,43] are inappropriate for such materials. For example, using the pin-on-disk wear test for a TSDC cutting element, there was no measurable weight loss of the sample and no detectable wear flat produced. The SEM-SEI image (Figure 9) of the tip of the sample shows not observable wear of the diamond composite material.

One recent approach to this issue of low abrasive wear was tested using single point PCD cutting tools with WC (13% Co) and Duralcan with 20% SiC as the workpieces [44]. The emphasis in the testing was low cost and rapid turnaround time and was especially geared to machining operations of these engineering materials. 

A more abrasive wear test procedure has been devised by CSIRO that spans the range from hard to superhard tool materials [45]. In these tests, a vitrified bonded alumina grinding wheel of standard manufacture is used as the workpiece. The cutting elements in these tests were solid, moulded TSDC samples and diamond composite coated tools on a WC substrate. In the latter case, the diamond composite was a PCD type and hence not thermally stable. The alumina grinding wheel was chosen in preference to the more abrasive SiC grinding wheel because of the requirement to cover WC cutting tools, diamond composite as well as cubic boron nitride tools. The properties of the alumina grinding wheel and test conditions were:

- Density:      2.18 g/cc
- Compressive strength:  121 MPa
- Tensile strength:   20 MPa
- Cerchar Abrasivity Index (CAI): 3.54
- Porosity:    ~42 vol %
- Al_2_O_3_ grain size:   ~400 microns
- Wheel dimensions:   350mm OD × 51mm width
- Rotational speed:   480 rpm
- Depth of cut:    ~0.3mm
- Feed rate:    ~1.2mm
- Testing conditions:   Dry cutting

**Figure 10:**
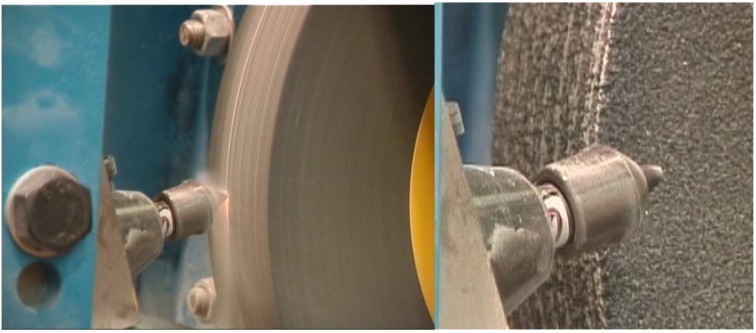
Photos of the CSIRO’s abrasive wear testing rig for diamond composite cutting elements with an alumina grinding wheel as the counter piece for the test. Note: the high temperature region in the vicinity of the cutting tip (left) and tool holder (right).

**Figure 11:**
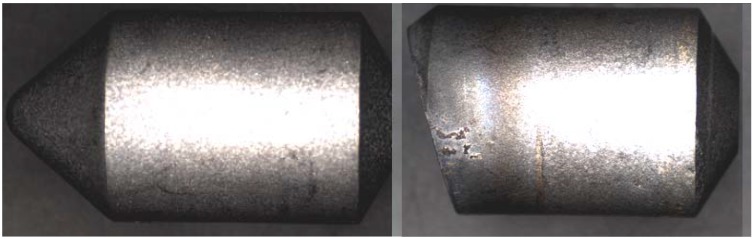
Cemented tungsten carbide cutting element (WC) before (left) and after only 20 cuts on the abrasive wear testing rig (right).

**Figure 12:**
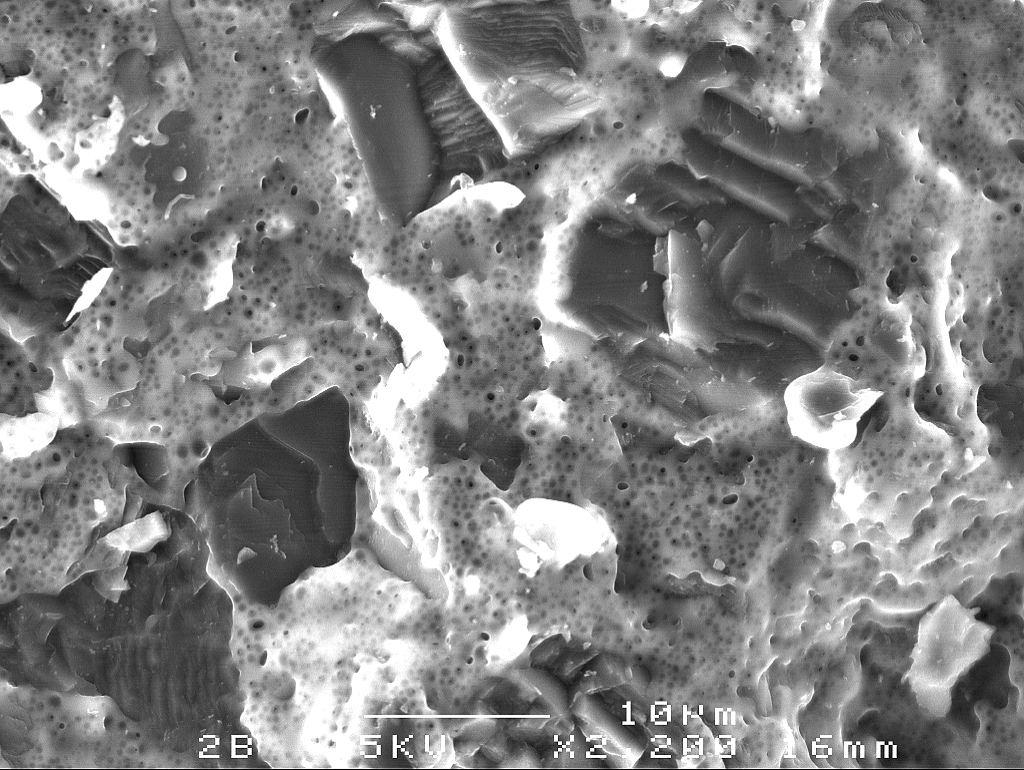
SEM-SEI image of a poor quality TSDC cutting element (see M2-b, Table 1). The diamond grains are clearly delineated with cleavage fracture features while the matrix is unreacted silicon metal with the porous microstructure. The bonding of the diamond to the unreacted Si appears to be weak as there is evidenced by the diamond grains having been plucked out during the wear test.

The wheel was “machined” across its face (Figure 10) for a set number of traverses which, for the TSDC cutting elements, was 100. Depending on the wheel diameter this could amount to cumulative cutting distances of over 4000m. However, for poor performing tools the number of cuts had to be limited either because the tool had worn excessively as in the case for WC (Figure 11) or because the diamond composite was of poor quality and the tool wore through to the underlying carbide substrate (Figure 12).

Two levels of tests have been used in CSIRO’s wear testing studies. Level one is that specified in an earlier study [45] in which cumulative wear rates, dimensional wear coefficients (*k*) and specific energies may be calculated while level two consists in measuring the weight loss of the cutting element to the nearest 0.0001 g and the volume or weight loss of the work piece during the test. These simple data yield the empirical wear rate - loss of weight of the cutting element (grams) per unit volume cut from work piece (m^3^) or the empirical wear resistance (viewed as a figure-of-merit) which is the weight of material cut per unit weight loss of cutting tool—either measure may be used as a quality factor for assessing the performance of diamond composite cutting elements. Any cutting element can be assessed by this latter method, once the tool holder has been modified to accept the shape of the element. The results of a wide ranging study of diamond composite cutting elements from various manufacturers are presented in Table 1, Table 2 and Table 3. (Note: no individual manufacturer has been identified in these tests as it is not the purpose of the study to rank the quality of the manufacturers, only the quality of their products. The microstructural investigations have been specifically directed at determining the possible causes for the poor wear performance of the various grades of diamond composites used.)

**Table 1:** Wear of ballistic ^1^ shaped TSDC cutting elements.

Element ID	Wear Rate (g/m^3^)	Wear Resistance (×10^3^) (wt material cut/wt loss tool)	Depth of Cut (mm)	Length of Cut (m)
M1-a	12.0	183.6	0.27	4181
M2-a	53.0	41.5	0.29	4197
M2-b	256.1	8.6	0.18	883
M2-c	6.9	318.8	0.28	4211
M3-a	4.8	458.6	0.27	3478
M3-b	111.4	19.7	0.24	4265
M3-c	3102.5	0.7	0.05	365
M3-d	5.1	432.0	0.26	3508
M3-e	4.3	516.9	0.27	4222
M3-f	3.6	607.0	0.29	4193
M4-a	19.7	111.7	0.26	4215
M5-a	18.1	121.4	0.38	3152
M6-a	8.4	261.1	0.27	2197
M6-b	2.9	749.2	0.29	3854
M6-c	13.4	164.4	0.29	4193
M7-a	232.3	9.5	0.15	404
M7-b	1960.6	1.1	0.08	375
M7-c	4765.2	0.5	0.08	410
M7-d	6865.8	0.3	0.10	905

Note: variations in the depth of cut arise from the relative wear rates of the cutting element, the poor quality tools are not able to maintain the set depth of cut. 1 the precise shape of the ballistic cutting element is subject to “commercial-in-confidence” agreement.

**Table 2:** Wear of cylindrical cutting elements.

Element ID	Wear Rate (g/m^3^)	Wear Resistance (×10^3^) (wt material cut/wt loss tool)	Depth of Cut (mm)	Length of Cut (m)
M2-d^1^	2.7	818.9	0.28	4206
M7-e^2^	6.3	351.0	0.29	3482
M8-a^2^	10.3	213.7	0.29	3511
M9-a^3^	14.2	155.3	0.26	4227

Note: 1. solid TSDC; 2. PCD layer on WC substrate; 3. solid TSDC with 98% diamond.

**Table 3:** Wear on ballistic^1^ shaped cutting elements - PCD coating on WC.

Element ID	Wear Rate (g/m^3^)	Wear Resistance (×10^3^) (wt material cut/wt loss tool)	Depth of Cut (mm)	Length of Cut (m)
M3-g	4.9	448.3	0.30	3946
M5-b	3.1	716.8	0.29	2798

1 The precise shape of the ballistic cutting element is subject to “commercial-in-confidence” agreement.

Even within the category of diamond composite cutting elements, the wear resistance across several manufacturers can vary from 0.3 to 818.9 × 10^3^. For comparative purposes, the wear resistance of mining-grade WC was observed to vary between 0.01 and 0.12 × 10^3^. For the purposes of assessing the relative wear behaviour of hard and superhard cutting elements, CSIRO’s wear testing procedure is a simple and low cost operation.

### 5.1. Microstructural observations

#### 5.1.1. Solid TSDC

The CSIRO abrasive test methodology is not registered as a standard assessment procedure hence the results are only indicative of the relative wear behaviour of this class of superhard material. However, it is clear from the results shown in Table 1, Table 2 and Table 3 that the wear rates of solid TSDC samples cover an extremely wide range of values. It has yet to be determined what is an acceptable wear rate for this class of material but, based on the wear results, one may tentatively assign the qualitative terms such as “good wear resistance”, “low wear rate” or “good quality” to TSDC elements with wear rates less than 10 g/m^3^ (or equivalent wear resistances greater than ~ 200 × 10^3^), while samples with wear rates greater than this value as “poor quality”. Certainly, more research and cross-laboratory studies are required to determine more precisely the quality of these superhard materials and to relate wear test parameters to cutting performance of tools in field trials.

In an idealised microstructure, a TSDC element is expected to contain SiC as the binder phase, produced during the HPHT reactive bonding process. As the microstructural studies presented in Section 4 indicate, such an idealised mix of phases has not been observed. Certainly these phases are present in most samples but there are other phases such as residual silicon metal, graphite and a range of chemically distinct but as yet unidentified phases that occur in the matrix of these TSDC samples. The presence of such contaminant phases is the most likely cause of the variable wear resistance. For example, in TSDC sample M2-b (Table 1) with an unacceptably high wear rate of 256.1 g/m^3^, an SEM study of wear surface revealed that there were significant amounts of unreacted silicon metal in the microstructure, indicating that the reactive bonding process at the HPHT conditions of manufacture was incomplete—Figure 12.

**Figure 13:**
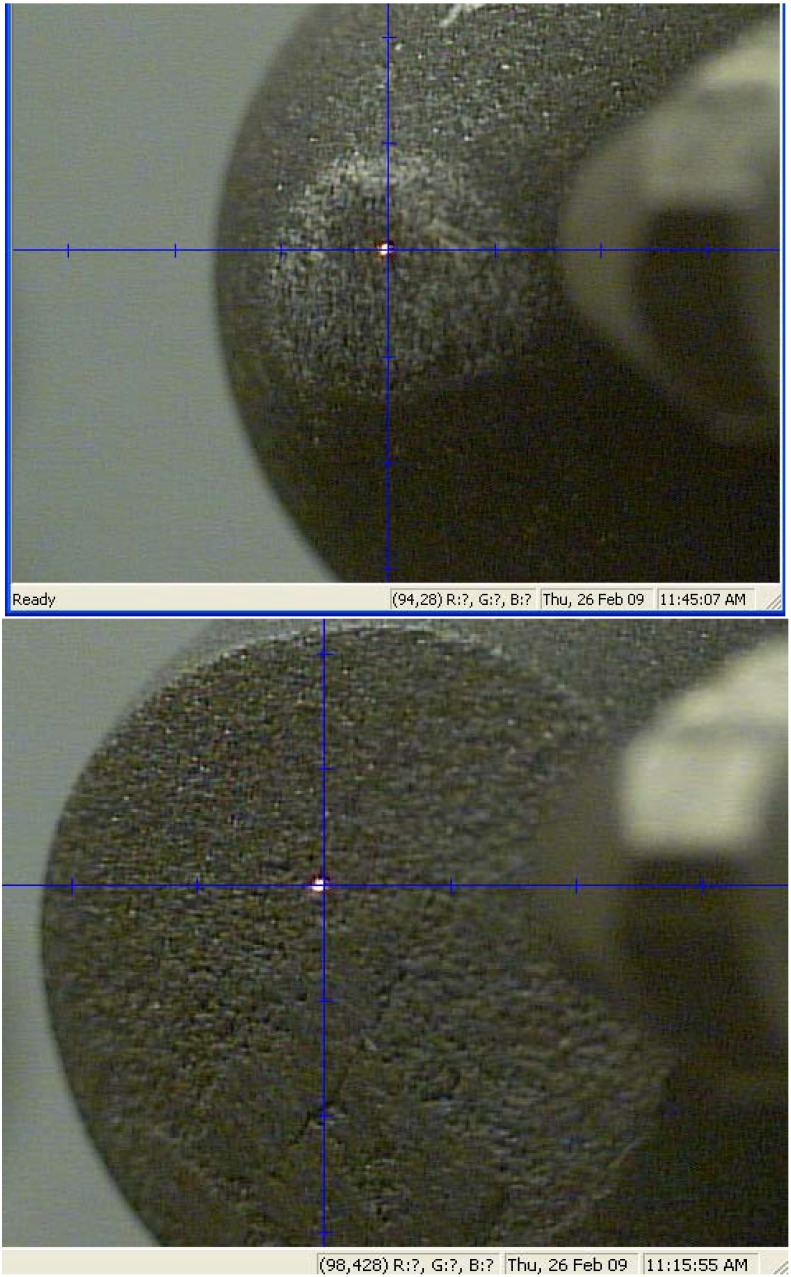
Optical images of both samples as mounted in the sample holder of GADDS^TM^. Upper image is of the high quality TSDC (*i.e.,* high wear resistance, sample 1) with a small wear flat (upper) and the lower image of poor quality TSDC (*i.e.,* low wear resistance, sample 5) showing the large wear flat. The bright laser spot on each of the wear surfaces indicates the location of the x-ray beam used in the analyses (Magnifications: upper image ×40, lower image ×30).

**Figure 14:**
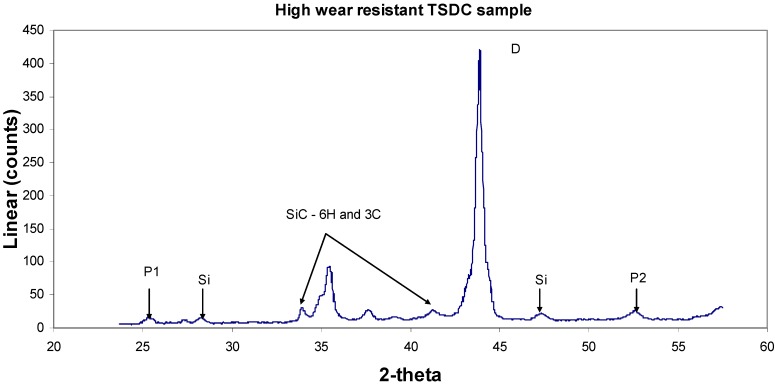
X-ray diffraction spectrum of sample 1 (good quality TSDC with high resistance). The automated peak search revealed diamond, silicon metal, the 6H and 3C polytypes of SiC with two unknown peaks, P1 and P2.

**Figure 15:**
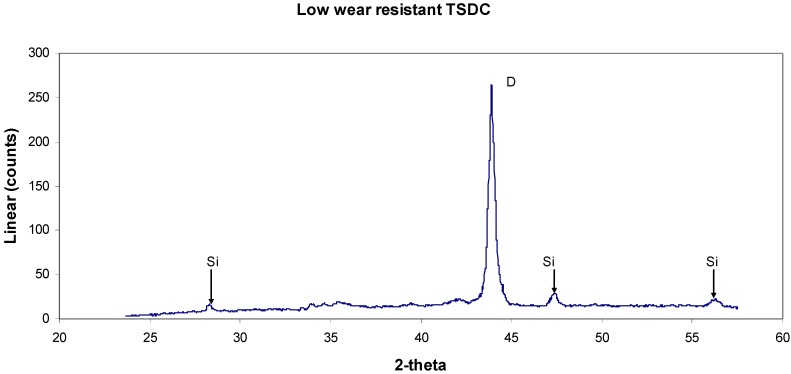
X-ray diffraction spectrum of sample 5 (poor quality TSDC) with diamond and Si metal positively identified. There are several smaller peaks between 33° and 43° that can tentatively assigned to the SiC polytypes—refer to XRD trace above - but appear to be in much smaller quantitatives than in sample 1.

**Figure 16:**
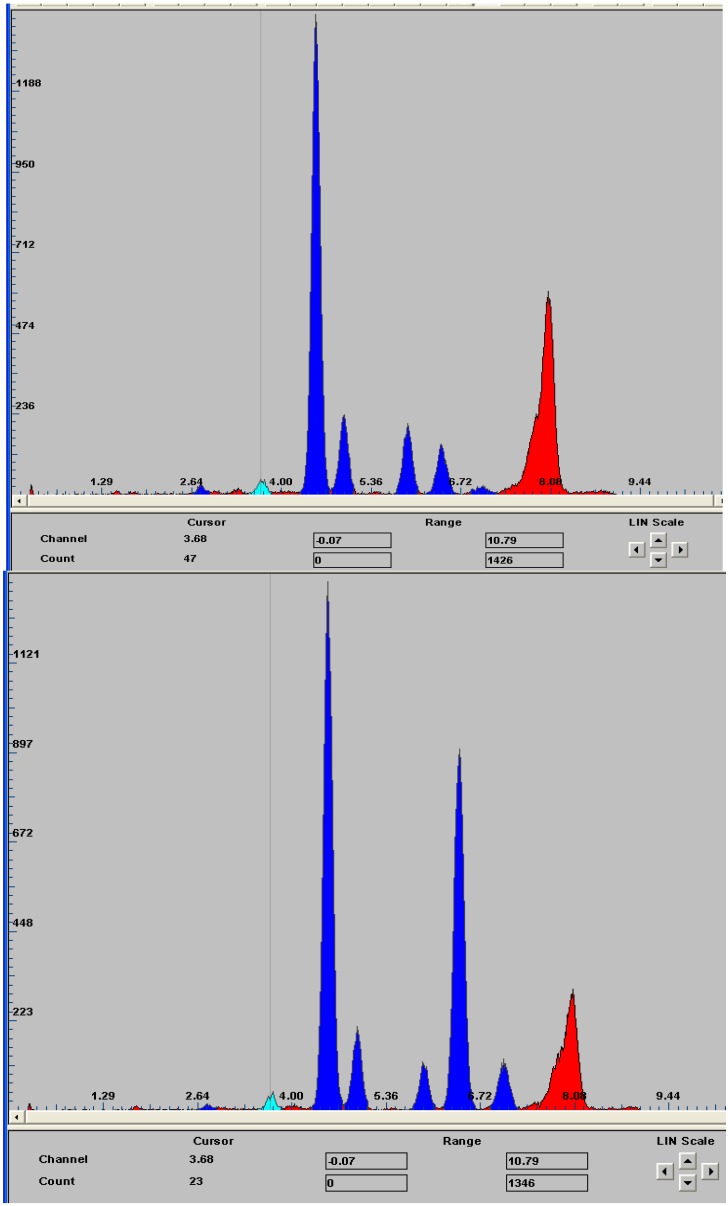
EDS spectra observed in the GADDS^TM^ analyses from Sample 1 (upper spectrum) and sample 5 (lower spectrum).

A detailed study of two solid TSDC samples (designed as samples 1 and 5) with markedly contrasting wear behaviour was undertaken using GADDS^TM^ (General Area Detector Diffraction System) with Cu K_α_ radiation (λ = 1.5418 Å). The major advantages of this system are: (1) no special surface preparation is required; (2) it is possible to maintain spatial integrity and hence examine the homogeneity of the phase distribution across the worn surface; (3) reasonably large-sized samples can be mounted in the specimen holder. The results from a study of the worn surfaces in these samples are shown in Figure 13, Figure 14, Figure 15 and Figure 16.

The phases and the sample chemistries vary greatly between these samples. Sample 1 shows x-ray diffraction evidence of hexagonal and cubic SiC polytypes as well as metallic silicon in addition to the strong diamond peak. There are two as yet unidentified peaks (P1 and P2) at 3.507 Å and 1.736 Å. The metallic Si is evidence of residual, unreacted Si as previously discovered in the SEM-EDS observations shown in Figure 4. Sample 5 has both the diamond and silicon metal with trace amounts of SiC as well as three unidentified peaks at 3.154, 1.917 and 1.635 Å. Perhaps the more significant data are shown in the EDS spectra of the two samples. Both samples have strong Ti peaks with the poor wear-resistant sample 5 containing significant Fe and Co contents (note that the broad peak around 8 keV is produced by the Cu Kα radiation source while there is poor sensitivity to X-ray photons at the low energy end of the spectra). The diamond concentration of these samples has yet to be determined.

#### 5.1.2. PCD coating on WC substrate

PDC/PCD-coated WC elements are used extensively in drill bits in the mining, exploration and civil construction industries [27,28,29]. Although the composite coatings may have varying thicknesses, the samples examined in this study have thickness of approximately 0.5 mm and are sintered onto a WC substrate under HPHT conditions within the diamond stability phase field—refer Figure 1. The coatings are limited to the upper section of the cutting element which may have either a conic shape with variable included angle or a proprietary ballistic shape. At present, the diamond composite used in coating the cutting elements is not classified as thermally stable, having a complex mix of phases, including cobalt, which is also the dominant binder phase used in the WC substrate. As with the solid TSDC cutting elements, these PCD-coated cutting elements exhibited an extremely wide range of wear resistance.

**Figure 17:**
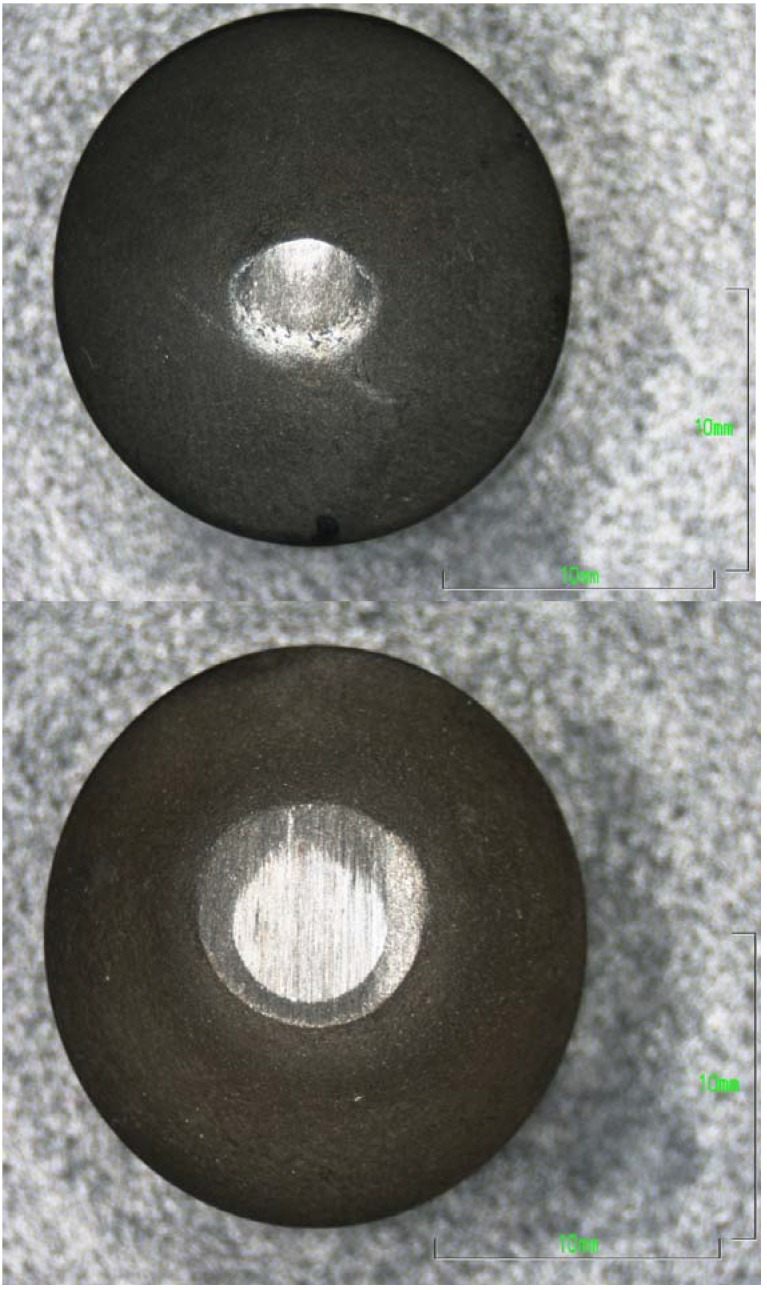
Top views of diamond coated cutting elements sintered onto a WC substrate: upper image showing the wear flat generated on a high quality sample after 100 cuts (approximate cutting distance of 3399 m and wear rate of 6 g/m^3^); lower image showing the excessive wear produced on a poor quality sample in which the diamond coating abraded through to expose the WC substrate after 4 cuts (~152 m) (scale markers: 10 mm).

The macrographs shown in Figure 17 are examples of the variability in quality of these coated samples. The upper sample was classified as good quality with a wear rate of ~6 g/m^3^ after 3399 m of abrasive wear on the corundum wheel—this sample has similar wear parameters to sample M2-c in Table 1. The small wear flat was totally contained within the diamond composite coating.

The lower sample in Figure 17 had such poor wear resistance that the diamond coating had worn through to the underlying WC substrate after only 152 m of abrasive wear on the corundum wheel—this corresponds to only 4 cuts across the face of the workpiece compared with 100 cuts in the good quality sample. In such cases, any measurement of weight loss of the sample would be heavily biased by the removal of some of the high density WC substrate. To run a valid test in which only the diamond composite coating is abraded away, two cuts or ~75 m of abrasive wear was the maximum distance. Even then, the wear rate was measured at 7800 g/m^3^—equivalent to M7-d in Table 1.

**Figure 18:**
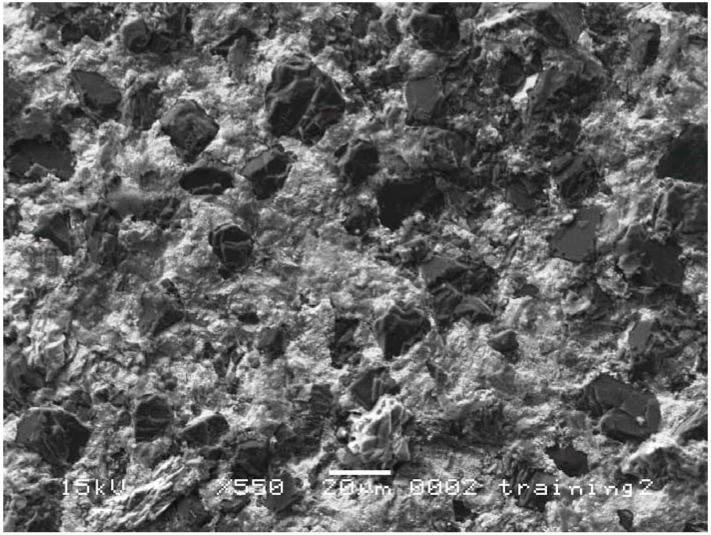
SEM-BEI image of the top worn surface of poor quality PCD-coated WC cutting element shown in Figure 17 (lower image).

**Figure 19:**
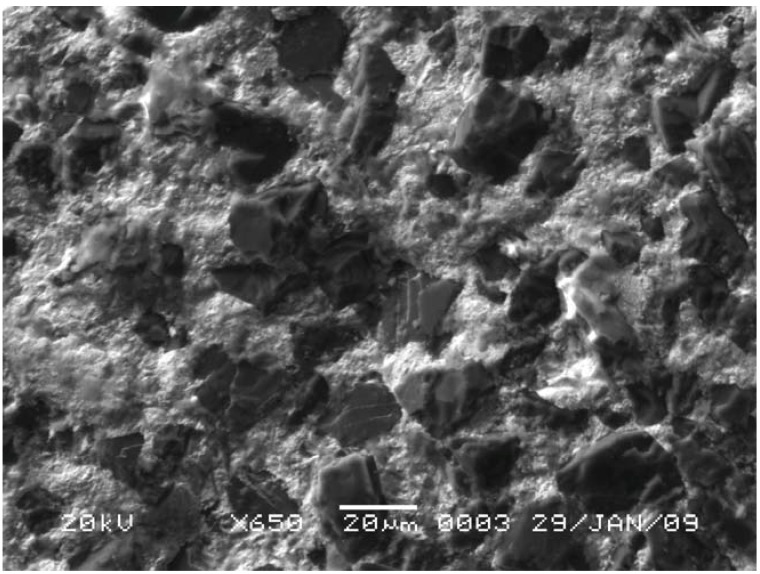
SEM-BEI image of top worn surface of good quality PCD-coated WC cutting element shown in Figure 17 (upper image).

Both samples were examined in the SEM (Figure 18 and 19) and an image analysis showed the only obvious difference between them is the volume percentage diamond (Table 4). Further investigation is underway to positively identify other significant differences between these two samples using EDS, XRD and Raman techniques.

**Table 4:** Average grain size and diamond content in PCD-WC cutting element.

ID	Average diamond grain size, microns	Volume % diamond
Poor quality	13.6	39
Good quality	15.1	57

#### 5.1.3. X-ray imaging

**Figure 20:**
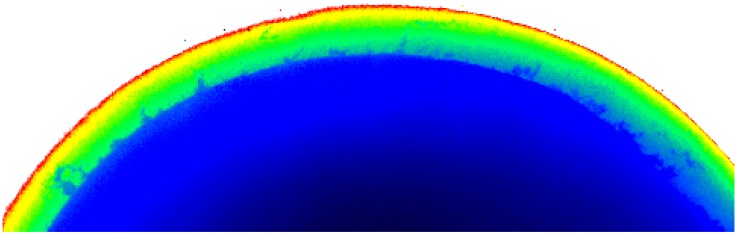
X-ray projection image of a PCD coating on WC substrate (blue region). Note the extent of intrusion of the WC substrate into the coating as well as the small cracks confined with the coating itself (scale: coating thickness ~454 μm.)

A very recent study has been initiated in which the quality of the diamond composite coating on WC is being assessed using X-ray imaging. The first set of images revealed, in somewhat surprising detail, the macroscopic defects generated in this form of coating (Figure 20). This is a projected image, generated from a point-focus X-ray source (70 keV, 80μA), of a PCD-coated WC sample sectioned longitudinally. Clearly, the interface between the coating and the WC substrate is rough with frequent intrusions of the WC substrate into cracks that were created at some stage in the manufacturing process. These cracks run almost to the outer surface of the sample. Such poor quality coatings would account for the high wear rates sometimes observed in coated samples.

Subsurface integrity of diamond coatings would be difficult to assess from optical and scanning microscopy. An X-ray micro-tomographic study could also be used on whole samples to generate 3D images of the coating, provided a sufficiently intense beam were available, such as a synchrotron source. Future research work will be undertaken to develop this technique.

### 5.2. Cutting mechanisms and wear of diamond composite cutting elements

Diamond composite tooling can be employed in cutting a wide variety of materials. The most significant parameters to monitor are the wear rates of the individual phases in the composite itself as these determine the useful lifetime of the tool. However, wear rates can also be influenced by the retention rate of the diamond cutting elements which in some operations is the individual diamond grains (referred to as grit by some tool manufacturers). To strengthen the bonding of the diamond grains in the matrix, some manufacturers pre-coat the diamond grit with carbide-forming metals such at Ti. This treatment has been reported to increase the life of the cutting tool, especially for diamond impregnated MMC tools [46].

**Figure 21:**
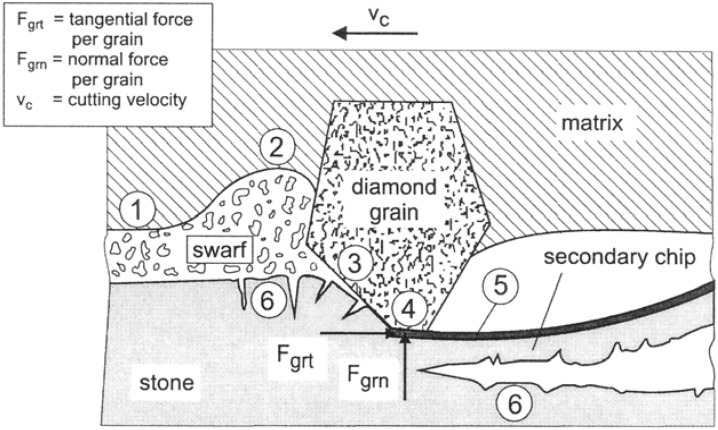
Microscopic model of the chip forming processes based on the interaction between an individual diamond grain and the work piece. This model is specific to brittle materials such a stone (Reproduced with permission of Trans Tech Publications – [52])

In order to understand the wear behaviour of diamond composite tools it is important to examine in detail both the chip formation process and the wear mechanisms. In the cutting of brittle materials, and especially rocks and stone, the dominant consideration has generally been the interaction between an individual diamond grain and the material surface. Most descriptive models envisage an individual diamond grain, embedded in the matrix of the tool, cutting into the work piece. With the diamond grain size ranging for 10 to 50 micron, the exposed portion of the active cutting grain is approximately half that value resulting in chip sizes in the micron to sub-micron size range [47,52]. Such a model is illustrated in Figure 21 for a diamond impregnated MMC segment cutting rock or stone. It is not intended to analysis this model in detail but it is clear that the chipping process is microscopic with submicroscopic fracture and fragmentation occurring in the work piece.

The major factors influencing cutting performance are:

- Quality of the diamond composite relative to abrasive wear mechanisms;
- The physical and chemical properties of the work piece—the latter aspect is vitally important in metal cutting although this area has purposefully been omitted from this review;
- Reaction forces on the tool;
- Stress distribution in both the tool and the work piece;
- Temperature at the tool—workpiece interface resulting from the frictional forces;
- Cutting speed and depth of cut;
- Removal of swath in the zone in front of the diamond grain.

A more innovative approach to the cutting operation in brittle materials, especially in the areas of rock and mineral excavation for mining and civil construction, is to use diamond composite cutting elements that will enable the depth of cut to be increased by at least two orders of magnitude. This approach has been adequately demonstrated in linear cutting operations [45] as well as in rotary cutting using a cutter drum. In the research studies presented in this review, the cutting element has been, in the first instance, TSDC. More recently this work has been extended to include cutting elements consisting of a diamond composite coating, generally PCD, on a cemented WC substrate. As discussed in Section 5.2.1, the PCD coating must be rated as non-thermally stable.

#### Phenomenological model of cutting brittle materials

**Figure 22:**
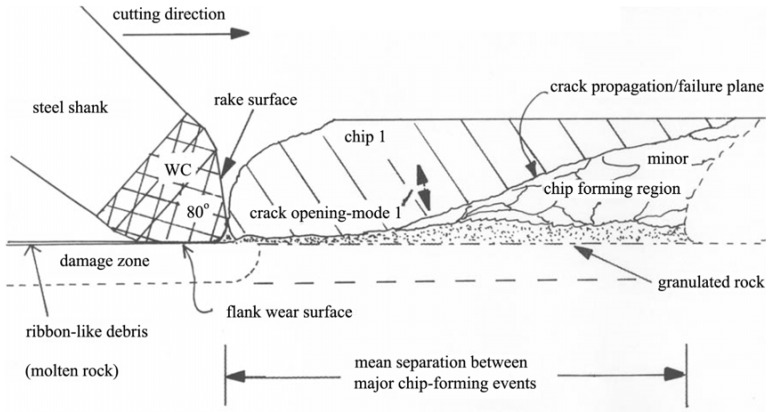
Phenomenological model for macro and micro chip formation in brittle materials such as rock.

The cutting operation can be arbitrarily partitioned into two forces acting on the work piece: the normal force required to maintain depth-of-cut and the cutting force. The effect of the normal force is equivalent to the action of an indenter with its accompanying deformation, especially the accompanying cracking and fracture under the indenter [48,49,50]. The chip formation mechanism consists in the propagation of a series of cracks initiated in the deformed volume surrounding the cutting element (Figure 22). Using high speed photography, it has been observed that the first detectable movement of macro chips corresponds to a tensile opening mode of fracture (mode I—see [48]), followed by a sliding of the separated chip across the fracture plane. Using an optical pyrometer, the temperature on the rock surface immediately behind the cutting tool can rise to ~1350 °C—these observations were made using cemented WC cutting elements [51].

Using this phenomenological model of chip formation, the more important aspects of the wear behaviour of diamond composite tools can be highlighted. Clearly, micromechanisms are the fundamental processes for both rock fracture and wear of the diamond composite. However, instead of microchip formation at the front surface of individual diamond grains with the concomitant gauging out of the binder phase directly ahead of the grain [47,52], somewhat less severe abrasive/erosive mechanisms occur on the rake and flank surfaces of the diamond composite cutting element. Further, the normal force will be distributed over the larger flank surface of the diamond composite cutting element. This force distribution contrasts greatly with the resultant forces expected on individual grains as envisaged in the micro chipping process for diamond impregnated MMC cutting elements. A more detailed analysis of this cutting model is the subject of ongoing research.

## 6. Conclusion

The wear behaviour of commercially produced diamond composite tools is highly variable. Since one of the major operational areas for diamond composites is in cutting, drilling and sawing for the mining, exploration and civil construction industries, there is a need for a simple and cost effective abrasive wear test for these superhard tool materials. As a first assessment procedure, an appropriate wear test of the type developed at CSIRO is recommended.

In order to determine the source of any poor wear performance of diamond composite materials, it is also essential to study their microstructural development. . It has been shown in this review that optical microscopy, scanning electron microscopy and X-ray microscopy provide important data for assessing the homogeneity and phase integrity of diamond composites. These methods are supplemented by XRD, EDS and Raman spectroscopy. It appears that, for TSDC cutting elements, the most significant processes or microstructural features responsible of high wear rates can be any of the following:

- Incomplete reaction of the molten silicon to SiC in the HPHT reaction bonding process;
- Failure to quench in the HPHT phases, leading to back transformation of diamond to graphite—this reaction may be catalysed by the presence of contaminants such as Co, Ni or Fe used in the initial production of the synthetic diamonds;
- Residual graphitic material in the initial diamond charge used in the HPHT sintering process.

For the PCD-coated WC tools, the more likely wear mechanisms are:

- Delamination of the PCD coating due to weak bonding between the WC substrate and the coating;
- Premature abrasive wear accompanied by cracking at the macrostructural defects in the coating.

Based on these observations, there is clearly a need to improve the quality of TSDC and PCD-coated WC cutting elements by better quality control of the reactive sintering process. All poor quality components have both residual silicon metal and graphitic material in their microstructures. The presence of the former component indicates incomplete reactive sintering under the HPHT conditions, while the source of the graphite has yet to be determined. It is essential that the only remaining allotropic form of carbon in the cutting element is diamond.

Although not specifically covered in this study, x-ray photoelectron spectroscopy (XPS) in combination with Raman spectroscopy will also assist in deciphering the type of carbon bonds (sp^2^ in graphitic material and sp3 in diamond), and hence the crystalline structure of carbon in these microstructures. Finally, cathodoluminescence has been shown to elucidate the phase distribution in a range of diamond composites as well as providing a means of assessing the quality of the diamond phase itself.
